# Circ-NFKB1 sponges miR-203a-5p to regulate ERBB4 expression and promotes IL-1β induced chondrocytes apoptosis

**DOI:** 10.1186/s13018-023-03990-4

**Published:** 2023-07-25

**Authors:** Zhao Wang, Hongwei Bao, Jingzhao Hou, Bin Ju, Yong Ji

**Affiliations:** 1 Department of Orthopedics, Jingjiang People’s Hospital, Jingjiang, Jiangsu Province China; 2 Department of Medical Imaging, Jingjiang People’s Hospital, Jingjiang, Jiangsu Province China; 3 Department of General Surgery, Jingjiang People’s Hospital, 28 No, Zhongzhou Road, Jingjiang, Taizhou City, 214500 Jiangsu Province China

**Keywords:** Circ-NFKB1, miR-203a-5p, ERBB4, Osteoarthritis, Apoptosis

## Abstract

**Background:**

Osteoarthritis (OA) is a chronic disease of the bones and joints that commonly affects middle-aged and elderly individuals, characterized by the degeneration of articular cartilage and inflammation of the joints. The molecular mechanisms of OA urgently need to be further examined. Our study intended to uncover circ-NFKB1/miR-203a-5p/ERBB4 axis in regulating interleukin-1β (IL-1β) induced chondrocytes apoptosis.

**Methods:**

GSE178724, GSE79258 and GSE169077 were downloaded from Gene Expression Omibus (GEO) database and differentially expressed circRNAs, miRNAs and mRNAs were obtained by R software. Annexin V assay was used to determine cell apoptosis rate. ELISA was further performed to identify the inflammation response. Dual-luciferase reporter gene assay was conducted to examine the combination among circ-NFKB1, miR-203a-5p and ERBB4.

**Results:**

Our research demonstrated that circ-NFKB1 and ERBB4 were significantly upregulated through bioinformatic analysis. MiR-203a-5p was significantly downregulated through bioinformatic analysis. Silencing of circ-NFKB1 notably inhibited the IL-1β induced chondrocytes apoptosis and upregulated ERBB4 expression. Through prediction on bioinformatics analysis, miR-203a-5p was the target binding circ-NFKB1, and ERBB4 was the potential target of miR-203a-5p. Subsequently, these changes induced by the silencing of circ-NFKB1 were reversed upon addition of pcDNA/ERBB4.

**Conclusions:**

Silencing circ-NFKB1 could sponge miR-203a-5p to regulate ERBB4 expression and alleviate OA progression.

## Background

Osteoarthritis (OA) is a chronic, progressive, and debilitating disease that results from cartilage degeneration, leading to the formation of osteophytes, thickening of subchondral bone, inflammation of the synovial membrane, damage to the meniscus, and degeneration of ligaments [1, 2]. Currently, more than 500 million people worldwide are affected by the impact of OA, with the peak incidence occurring around the age of 75 [3, 4].

Multiple factors, including age, sex, obesity, genetics, metabolic environment, and joint alignment, can lead to OA [5]. However, the exact molecular mechanisms that regulate the pathogenesis of OA remain elusive [6]. Apart from surgery, there are no effective interventions, prevention strategies, or treatments available that can slow down or reverse the progress of OA [7, 8]. Chondrocytes are the only cells found in mature cartilage, and in the case of OA, they undergo changes in non-coding RNA, which leads to a multitude of pathological changes [9–11]. Therefore, it is essential to explore the pathophysiology and molecular regulation mechanisms of chondrocytes in OA.

Non-coding RNA, which includes long non-coding RNA (lncRNAs), small RNA (miRNAs), and circular RNA (circRNAs), can affect biological processes by modifying DNA structure, RNA transcription, and protein translation through their regulatory functions [12–16. Advancements in RNA technologies, such as sequencing and bioinformatics analysis, have led to an increasing discovery of circRNA, revealing the principles behind its formation and biological functions [17]. With growing evidence of its diverse functions, circRNA has garnered significant attention as a key regulator in various cellular processes, including cell proliferation, apoptosis, differentiation, and invasion [18, 19]. CircRNA exhibits several salient features, including stability, specificity, conservation, and generality. The circRNA/miRNA/mRNA axis is believed to play an important role in the progression of OA, with circRNAs potentially serving as miRNA sponges to suppress mRNA translation and inhibit the occurrence and development of OA [20]. For example, Ouyang et al. [21] revealed that circDHRS3 aggravates IL-1β-induced extracellular matrix degradation, apoptosis, and inflammatory response via mediating methyl CpG binding protein 2 (MECP2) expression. LI et al. [22] found that knockdown of circSOD2 may serve as an intervention strategy to alleviate OA progression through modulating miR-224-5p/PRDX3 signaling axis. Liu et al. [23] revealed that circ_0002715 promotes the development of osteoarthritis through regulating LXN by sponging miR-127-5p.

In this study, we firstly performed bioinformatic analysis and revealed that circ-NFKB1 was significantly upregulated in OA patients. Further in vivo and in vitro experiments were performed to reveal the mechanism of circ-NFKB1 for regulating OA progression.

## Materials and methods

### Candidate lncRNA identification

In this paper, circular RNA sequencing raw data (GSE178724) of four normal tissue and four OA tissues were downloaded from the GEO database (https://www.ncbi.nlm.nih.gov/geo/query/acc.cgi?acc=GSE178724). GEO dataset was based on the platform of 074301 Arraystar Human CircRNA microarray V2. MiRNA sequencing raw data (GSE79258) of two normal tissue and two OA tissues were downloaded from the GEO database (https://www.ncbi.nlm.nih.gov/geo/query/acc.cgi?acc=GSE79258). GEO dataset was based on the platform of miRCURY LNA microRNA Array, 7th generation, miRBase 20 (Gene ID version). MRNA sequencing raw data (GSE169077) of two normal tissue and two OA tissues were downloaded from the GEO database (https://www.ncbi.nlm.nih.gov/geo/query/acc.cgi?acc=GSE169077). The microarray data was normalized using Bioconductor's fRMA package in R and subsequently differentially expressed circRNAs, miRNAs and mRNAs were analyzed with the R limma package. CircRNAs, miRNAs and mRNAs were considered significant if their absolute log2 fold change exceeded 1 and their p value was adjusted to be less than 0.05. The R heatmap package (version 0.7.7) was used to generate a heatmap.

### Cell culture and treatment

CHON-001 cells were acquired from the ATCC and grown in DMEM (Thermo Fisher), supplemented with 10% FBS and 100 U/ml penicillin/streptomycin in humidified 5% CO_2_ at 37 ˚C. CHON-001 cells were treated with (10 ng/mL) for 72 h to establish OA injury model.

### Cell transfection

Small interfering RNA (siRNA) against circ-NFKB1 (si-circ-NFKB1) (5′- ATGGTGGTCGGGTGAGAGAGT-3′), siRNA negative control (si-NC) (5′- AGGACATGGTGGTCGGGTGAG-3′), miR-203a-5p mimic (miR-203a-5p mimic) (5′- GGACATGGTGGTCGGGTGAGA -3′), mimic negative control (mimic-NC) (5′- GTCGGGTGAGAGAGTGAGCGA -3′), miR-203a-5p inhibitor (miR-203a-5p inhibitor) (5′- GTGGTCGGGTGAGAGAGTGAG -3′) and inhibitor negative control (NC-inhibitor) (5′-TGGTGGTCGGGTGAGAGAGTG-3′) were generated from Genepharma (Shanghai, China). pcDNA-based ERBB4 overexpression vector was obtained from Genepharma (Shanghai, China).

At about 70% confluence, CHON-001 were transfected with si-circ-NFKB1, si-NC, miR-203a-5p mimic, miR-203a-5p inhibitor and corresponding NC by Lipofectamine™2000 (Invitrogen) to perform follow-up experiments.

### Quantitative real-time PCR

The total RNA was isolated with the TRIzol reagent (Invitrogen, USA) before being examined with the NanoDrop 2000 (Thermo Fisher, USA). One microgram of total RNA was synthesized to complementary DNA (cDNA) by Revert Aid Reverse Transcriptase (TaKaRa, Tokyo, Japan). For miRNA, the MirX miRNA 1st Strand Synthesis kit (Clontech, USA) was employed for the cDNA synthesis. The expressions of the circRNA, miRNA, and mRNA were assessed through qRT-PCR, which was accomplished with the SYBR qRT-PCR Master Mix (Vazyme, China) or miRNA qRT-PCR SYBR Kit (Clontech), as appropriate. GAPDH and U6 were employed as the internal controls. The primer sequences are shown in in Table 1.

**Table 1:** The sequences of the primers in this study

Primer	Sequences
Circ-NFKB1	Forward: 5′- GAGGATGGGATCTGCACTGT-3'
	Reverse: 5′- GCGAAACCTCCTCTTCCTG-3'
miR-203a-5p	Forward: 5′- GTGAAATGTTTAGGACCACTAG -3′
Collagen II	Forward: 5′- TGAGAGGTCTTCCTGGCAAA -3' Reverse: 5′- ATCACCTGGTTTCCCACCTT -3'
MMP1 MMP13 ERBB4 GAPDH	Forward: 5′- GCCATATATGGACGTT-3' Reverse: 5′- CACTTCTCCCCGAATCGT-3' Forward: 5′- GCCAGATGGGTTTTGAGAC-3' Reverse: 5′-GTGATGCCTGGGGACTGTT-3′ Forward: 5′-TAGACCCGGGAGAAGGAAGAGCATC-3' Reverse: 5′-TCGCCCGGGTTATGACACCACAGTATTCCG-3' Forward: 5'-CCACTCCTCCACCTTTGACG-3'
	Reverse: 5′-CCACCACCCTGTTGCTGTAG-3'
U6	Forward: 5′-GCGCGTCGTGAAGCGTTC-3'
	Reverse: 5′-GTGCAGGGTCCGAGGT-3'

### Western blotting

Using western blotting per our previous protocol, the protein levels associated with apoptosis and oxidative stress were detected. In brief, radioimmunoprecipitation assay (RIPA) lysis buffer (Beyotime, China) was used to lyse the cells to extract the total protein, which was quantified using a BCA Protein Assay Kit (Beyotime, China) following the protocols. SDS-PAGE was utilized for separating the protein extracts, which were then blotted onto polyvinylidene difluoride (PVDF) membranes (Millipore, Bedford, USA) and blocked with 5% non-fat dried milk. Subsequently, the membranes were incubated at 4 °C with specific primary antibodies for 12 h, succeeded by 1 h of second incubation with the horseradish peroxidase-conjugated secondary antibody at room temperature. The concentrations of the primary antibodies for each antibody were as follows: caspase-3 primary antibody (ab32351, Abcam, Cambridge, UK, 1:1000 dilution), Bax primary antibody (ab32503, Abcam, Cambridge, UK, 1:2000 dilution), Bcl-2 primary antibody (ab182858, Abcam, Cambridge, UK, 1:2000 dilution), collagen II primary antibody (ab307674, Abcam, Cambridge, UK, 1:1000 dilution), MMP1 primary antibody (ab52631, Abcam, Cambridge, UK, 1:1000 dilution), MMP13 primary antibody (ab39012, Abcam, Cambridge, UK, 1:1000 dilution) ERBB4 primary antibody (ab19391, Abcam, Cambridge, UK, 1:200 dilution) and GAPDH primary antibody (ab9485, Abcam, Cambridge, UK, 1:2000 dilution). The protein bands were visualized with a western chemiluminescent ECL reagent (Tiangen, China) and quantified using ImageJ software.

### Bioinformatics analysis

CircInteractome (https://circinteractome.irp.nia.nih.gov/index.html) was used to predict the potential binding sites between miR-203a-5p and circ-NFKB1. TargetScan Human v7.2 (http://www.targetscan.org/) was used to predict the targeted binding sites between miR-203a-5p and ERBB4.

### Dual-luciferase reporter assay

The relationship between miR-203a-5p and NFKB1 or ERBB4 was investigated using Targetscan database. The NFKB1 luciferase reporter gene plasmids were utilized to demonstrate the potential targets of miR-203a-5p and NFKB1. The findings indicated that miR-203a-5p is a potential target of NFKB1. To conduct the reporter activity assay, Lipofectamine 2000 (Invitrogen) was used to co-transfect NFKB1 wild-type or mutant plasmids with miR-203a-5p mimic or mimic control into 293 T cells, following the protocol for 24 h. The luciferase activity was assessed using the Dual-Luciferase Reporter Assay System (Promega).

### RNA immunoprecipitation (RIP) assay

The EZ-Magna RIP Kit (Millipore, USA) was used to perform the RIP assay. In brief, chondrocytes that were cultured were collected and resuspended in RIP lysis buffer (Solarbio, Beijing, China). Afterward, the cell lysate was incubated with magnetic beads conjugated with Ago2 antibody (Abcam, USA) or IgG antibody (Abcam, USA) in RIP buffer overnight at 4 °C. The next day, the magnetic beads were washed three times with wash buffer and incubated with proteinase K. Co-precipitated RNAs were subsequently determined through RT-qPCR analysis.

### Annexin V assay

Detecting CHON-001 cell apoptosis was done via an Annexin V assay by an annexin fluorescein isothiocyanate (FITC) apoptosis detection kit (BD Biosciences, Franklin Lakes, NJ) and flow cytometry. In brief, after IL-1β culture in circ-NFKB1 presence or absence, the collected cells were resuspended in 500 μL binding buffer, which incubated then with 5 μL of each annexin V-FITC and propidium iodide (PI) in 15-min darkness at room temperature; eventually, a flow cytometer was utilized to analyze the cells. The cell death percentage was defined as PI and annexin V percentage summation.

### ELISA assay

After transfected for 24 h or treated with IL-1β for 12 h, the supernatant was collected from CHON-001 cells and the secretion levels of IL-6, IL-8 and TNF-α in supernatant were detected by ELISA kits (BD biosciences) following the protocol. Then, the optical density (OD) value at 450 nm was read on Multiscan Spectrum (MD, USA).

### Rat model of OA

Twenty Sprague–Dawley rats (9 weeks old, weighing 250–300 g) were randomly divided into four groups: the sham group, the OA group, the OA + adenoviral vector (AVV) siRNA-NC group and the OA + AAV siRNA-circ-NFKB1 group (5 rats/group). A total of 100 μl solution containing experimental or control virus (approximately 1*10E13 vg/ml) AAV siRNA-circ-NFKB1 was slowly injected into knees. The injection procedure was repeated after 4 weeks. The experimental protocols were strictly implemented following the guidelines outlined in the National Institute of Health's Guide for the Care and Use of Laboratory Animals. A model of OA was induced by anterior cruciate ligament transection (ACLT) as described by Yoshioka et al.^24^. Briefly, a medial parapatellar incision was made and an arthrotomy was performed. The patella was dislocated laterally and the knee placed in full flexion. The Anterior Cruciate Ligament (ACL) was visualized and transected with a No.12 blade. An anterior drawing test was performed gently to confirm that the ACL was transected completely. The joint was irrigated with sterile saline and closed. A sham operation was performed in the contralateral knee. The knee was opened, and the patella was dislocated. After performing the anterior drawing test gently, the joint was irrigated and closed. After the operation, free activity was allowed in the cage without immobilization.

Following administration of pentobarbital sodium anesthesia, an incision was made inside the joint capsule, which was medial and anterior to the collateral ligament. The anterior cruciate ligament was cut and the medial meniscus was resected. Following the procedure, the joint was irrigated with saline and the skin was sutured. In the sham group of rats, the incision on the inner side of the joint capsule exposed the anterior cruciate ligament but did not involve cutting it, and the medial meniscus was left intact.

### Hematoxylin and eosin (H&E) staining

The joint specimen was fixed in 10% neutral formalin for 72 h, followed by rinsing with PBS solution. Subsequently, specimen was soaked in EDTA decalcification solution for 3 months, with weekly replacement of the decalcification solution. After decalcification, it was rinsed with running water for 10 min and sequentially placed in 50% ethanol for 2 h, 75% ethanol for 2 h, 95% ethanol for 2 h, absolute ethanol I for 2 h, absolute ethanol II for 2 h, xylene I for 30 min, xylene II for 30 min, 65℃ paraffin solution I for 1 h, 65℃ paraffin solution II for 1 h, and finally embedded in paraffin using a paraffin embedding machine. The embedded specimens were sliced into 4 μm sections using a microtome, scooped out, and baked. They were then kept overnight in a constant temperature oven at 65 °C. The sections were dewaxed, rehydrated, and stained with hematoxylin for 5 min. After shaking off the excess stain, the sections were treated with differentiation solution for 30 s, followed by rinsing with tap water until a blue color appeared. Eosin solution was then added dropwise for 2 min, and the sections were rinsed with tap water for 10 min. Finally, the sections were dehydrated, cleared, mounted with neutral gum, and covered with a cover glass.

### Statistical analysis

All the experiments were repeated at least thrice, expressing the data as means ± standard deviation (SD). The mean comparison of multiple groups was carried out by one-way analysis of variance (ANOVA), while of two different groups was by independent-sample t-tests, performing all the statistical analysis with SPSS 20.0 (IBM Corp., Armonk, NY, USA), with *P* < 0.05 as a significant difference.

## Results

### Bioinformatic analysis

**Fig. 1:**
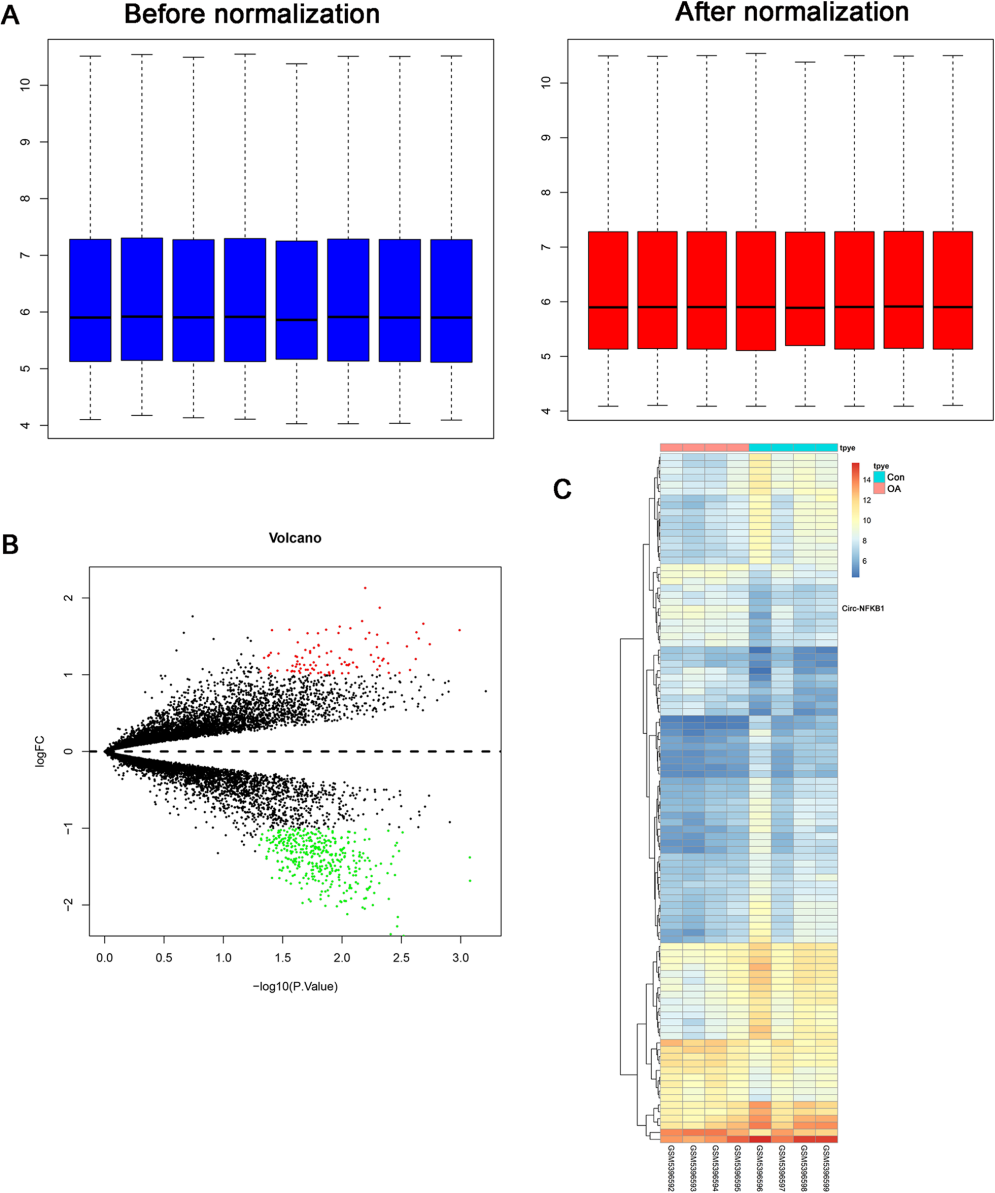
Differentially expressed circRNAs expression in OA and normal cartilage was analyzed using the GEO datasets (GSE178724). **A** Comparison of expression value between before normalization and after normalization. **B** A volcano plot of differentially expressed circRNAs between control and OA group. **C** Heatmap plot of top 100 differentially expressed circRNAs

**Fig. 2:**
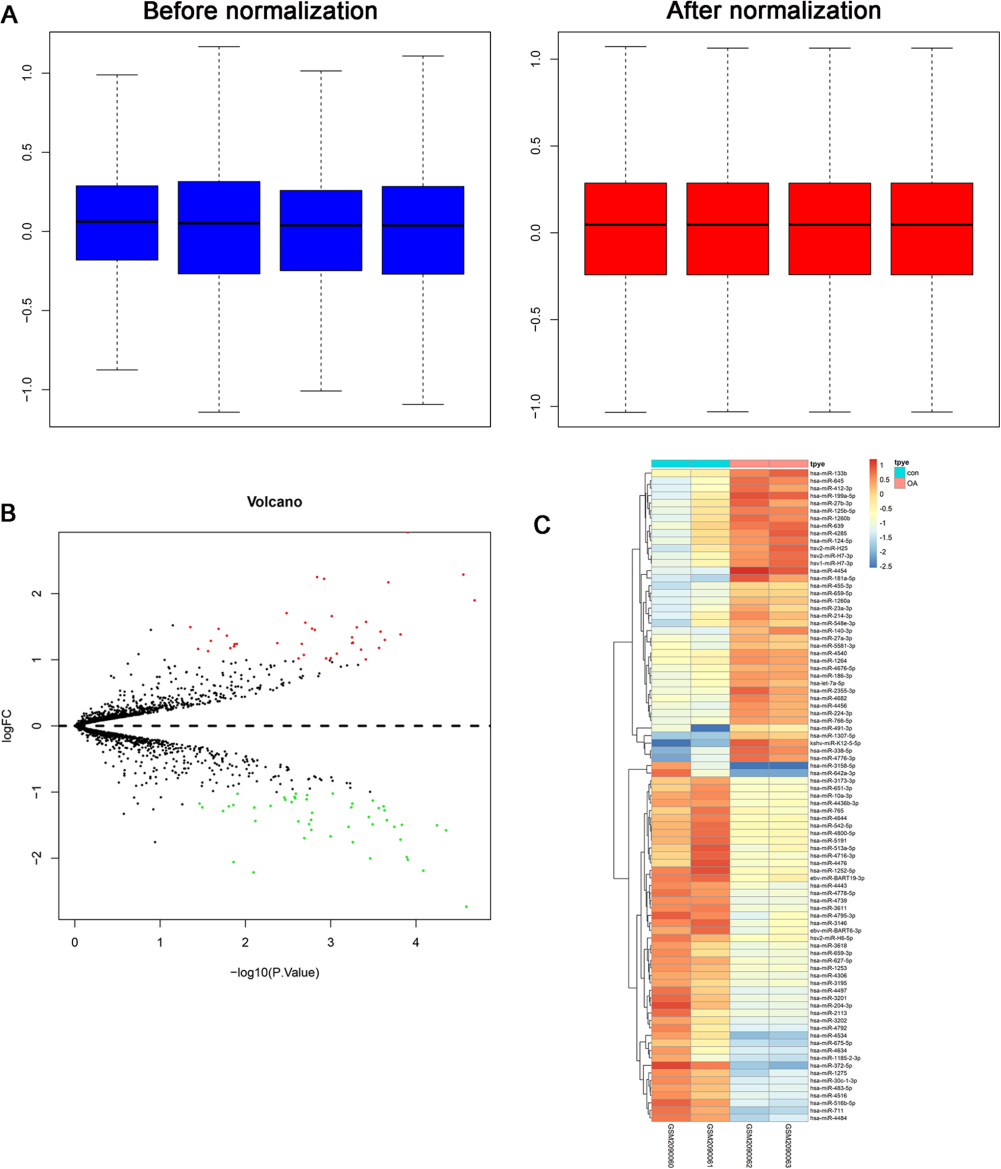
Differentially expressed miRNAs expression in OA and normal cartilage was analyzed using the GEO datasets (GSE79258). **A** Comparison of expression value between before normalization and after normalization. **B** A volcano plot of differentially expressed miRNAs between control and OA group. **C** Heatmap plot of differentially expressed miRNAs

**Fig. 3:**
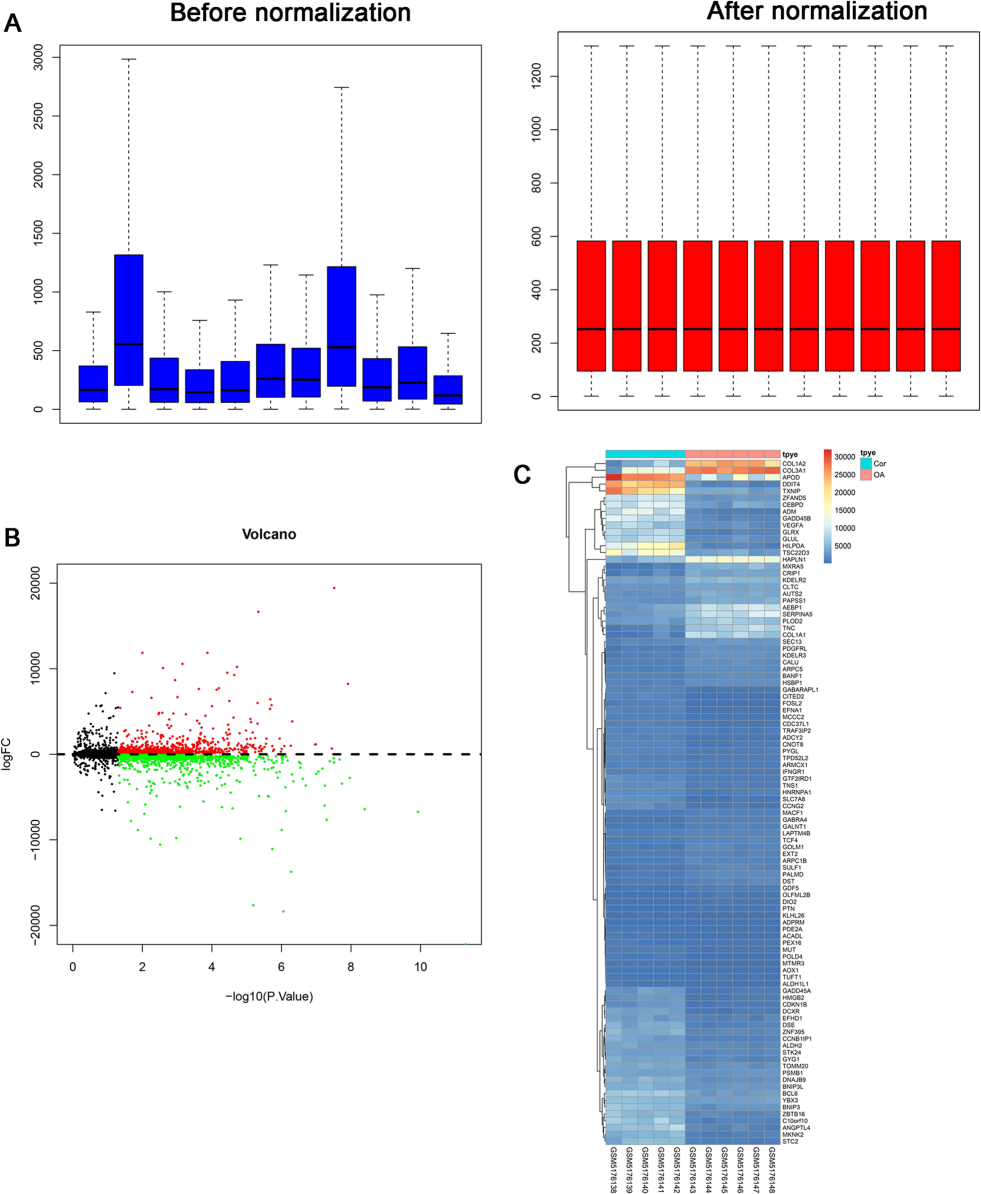
Differentially expressed mRNAs expression in OA and normal cartilage was analyzed using the GEO datasets (GSE169077). **A** Comparison of expression value between before normalization and after normalization. **B** A volcano plot of differentially expressed mRNAs between control and OA group. **C** Heatmap plot of top 100 differentially expressed mRNAs

After normalization, Fig. 1A depicts that the log2 ratios in the three pairs of samples are almost identical. A total of 484 differentially expressed circRNAs were identified, among which, 98 were upregulated and 386 were down-regulated (Fig. 1B). The top 100 most differentially expressed circRNAs were listed in Fig. 1C. We focused on circ-NFKB1 as top-ranked upregulated circRNA in OA as indicated by a significant P-value. After normalization, Fig. 2A depicts that the log2 ratios in the three pairs of samples are almost identical. A total of 87 differentially expressed miRNAs were identified, among which, 39 were upregulated and 48 were down-regulated (Fig. 2B). The differentially expressed miRNAs were listed in Fig. 2C. After normalization, the expression values were identical and could be used for further study (Fig. 3A). A total of 2643 differentially expressed genes were identified, including 1249 upregulated genes and 1394 downregulated genes. A total of 2643 differentially expressed mRNAs were selected according to the criteria (Fig. 3B and C).

### Circ-NFKB1 was induced in osteogenic differentiation

**Fig. 4:**
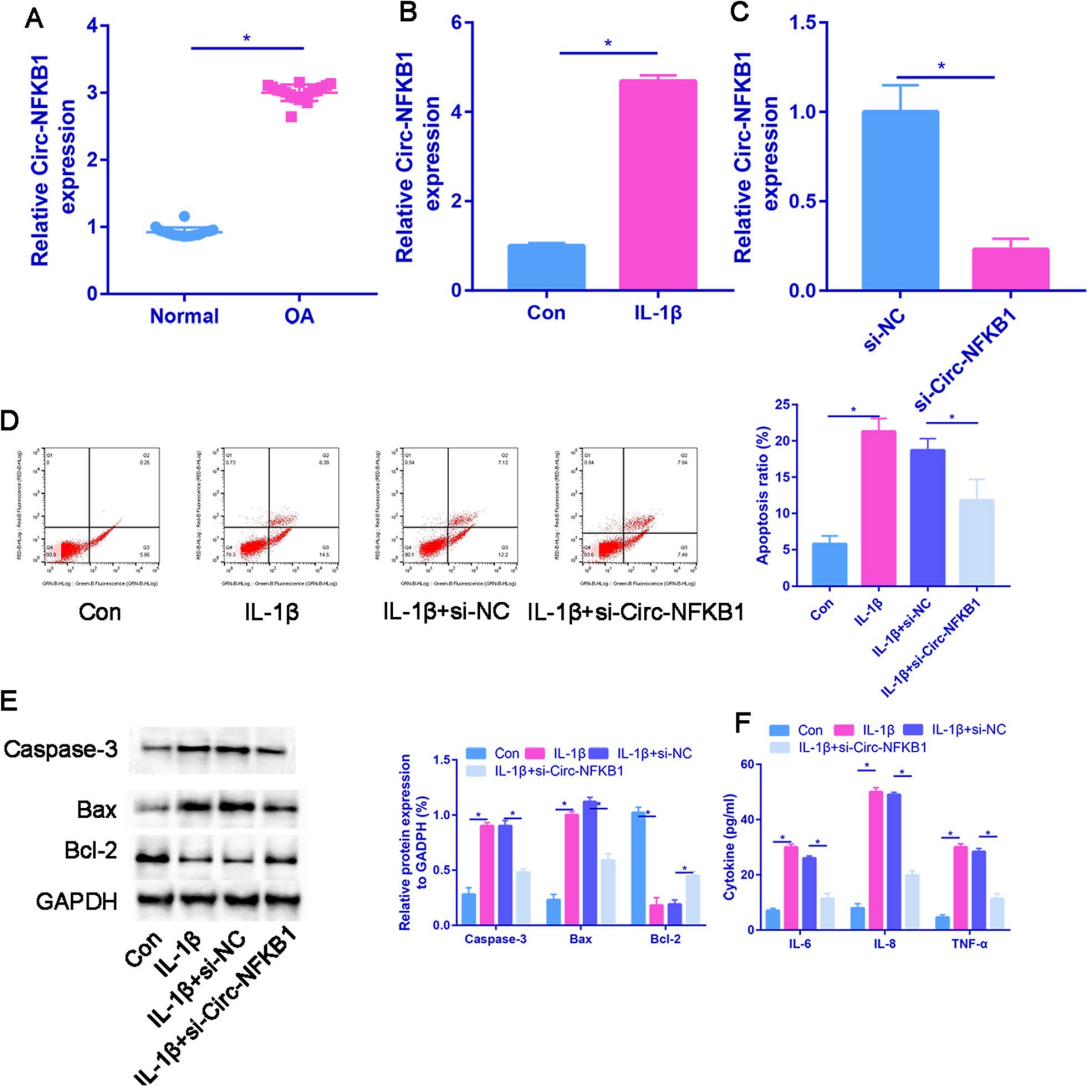
circ-NFKB1 is upregulated in OA cartilage tissues and chondrocytes. **A** The expression of circ-NFKB1 was measured in OA cartilage tissues and normal controls by RT-PCR analysis. **B** circ-NFKB1 level was detected in normal chondrocytes and IL-β-induced chondrocytes. **C** Relative circ-NFKB1 expression in si-NC and si-circ-NFKB1 groups. **D** Flow cytometer analysis for the apoptosis of chondrocytes: control, IL-1β, IL-1β + si-NC, IL-1β + si-circ-NFKB1. **E** Expressions of Caspase-3, Bax and Bcl-2 proteins detected by Western blot assay. **F** Changes in inflammatory cytokines in control, IL-1β, IL-1β + si-NC and IL-1β + si-circ-NFKB1 groups. *P < 0.05

In the RT‐qPCR analysis, circ-NFKB1 mRNA expression was saliently increased in OA samples compared with the normal group (Fig. 4A). Moreover, circ-NFKB1 mRNA expression was significantly increased in IL-1β treated chondrocytes than control group (Fig. 4B). Subsequently, chondrocytes were exposed to si-NC or si-circ-NFKB1. The circ-NFKB1 expression level was significantly downregulated in si-circ-NFKB1 treated chondrocytes (Fig. 4C). As shown in Fig. 4D, chondrocytes apoptotic ratio was significantly upregulated in IL-1β treated chondrocytes. While, si-circ-NFKB1 could partially decreased the apoptotic ratio of chondrocytes than IL-1β group (Fig. 4E). The si-circ-NFKB1 treatment reduced the levels of pro-apoptotic proteins, such as caspase-3 and Bax, while increasing the levels of the anti-apoptotic protein Bcl-2. This suggests that si-circ-NFKB1 has a protective effect against IL-1β-induced apoptosis. The concentrations of IL-6, IL-8, and TNF-α were measured via ELISA, revealing a significant increase in their levels in IL-1β-treated chondrocytes. However, treatment with si-circ-NFKB1 markedly decreased the levels of IL-6, IL-8, and TNF-α compared to the IL-1β group (*P* < 0.05, Fig. 4F).

### Downregulation of circ-NFKB1 alleviates OA development in vivo

**Fig. 5:**
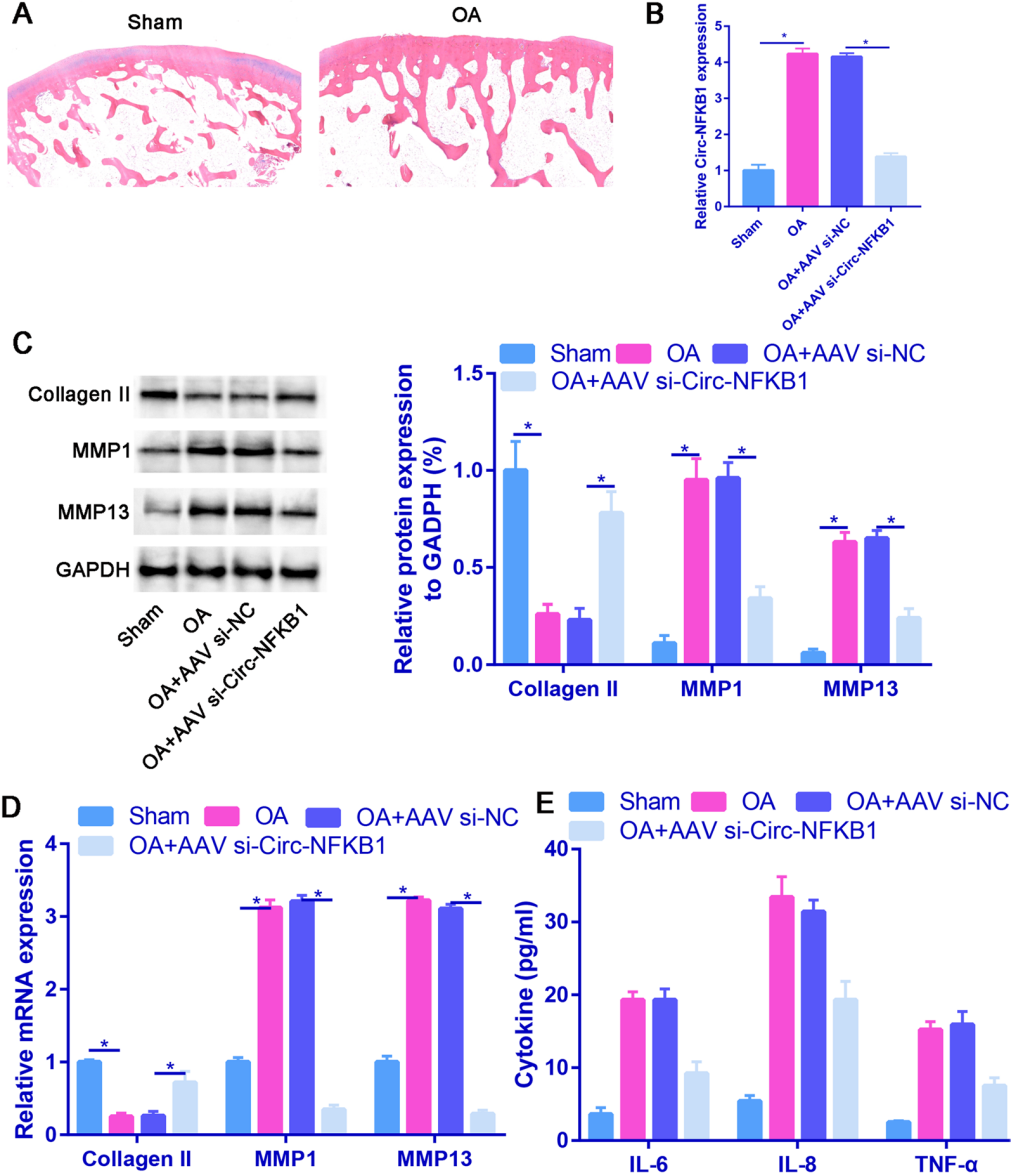
Downregulation of circ-NFKB1 alleviates OA development in vivo. **A** Pathological changes of knee joint from rats were observed using HE staining assay. **B** The expression of circ-NFKB1 was detected in cartilage tissue in sham, OA, OA + AAV-si-NC and OA + AAV-si-circ-NFKB1 rats. **C** The protein level of collagen II, MMP-1 and MMP13 were detected by western blot after injection of AAV siRNA circ-NFKB1 in OA rat model. **D** The mRNA level of collagen II, MMP-1 and MMP13 were detected by western blot after injection of AAV siRNA circ-NFKB1 in OA rat model. **E** ELISA was applied to determine the concentration of inflammatory cytokines (IL-6, IL-8 and TNF-α) in OA rats. *P < 0.05

In order to further investigate the impact of circ-NFKB1 on OA development in vivo, we successfully established an OA rat model, as depicted in the Fig. 5A. The cartilage surfaces of sham rats appeared smooth, with a clear depiction of the four-layered cartilage structure and the tide line. Conversely, OA rats cartilage surface exhibited irregularities, with small cracks and ruptures present. There is poor recognition of the tide line, and the depiction of the four-layered cartilage structure is not clear. As shown in Fig. 5B, the circ-NFKB1 expression level in OA samples was significantly upregulated than that of sham samples. Moreover, the injection of AAV si-circ-NFKB1 significantly reduced the expression of circ-NFKB1 compared to the OA + AAV si-NC group. Our findings revealed that the mRNA and protein levels of MMP1 and MMP13 were significantly increased in the OA rat model. However, injection of AAV si-circ-NFKB1 significantly reduced the mRNA and protein levels of MMP1 and MMP13 compared to the OA + AAV si-NC group. Interestingly, the protein expression of collagen II showed an opposite trend (Fig. 5C and D). Furthermore, the concentrations of proinflammatory cytokines, including IL-6, IL-8, and TNF-α, were notably increased in OA rats. However, circ-NFKB1 deficiency resulted in a significant decrease in the levels of pro-inflammatory cytokines in a rat model of OA (Fig. 5E). In conclusion, the downregulation of circ-NFKB1 attenuates OA development by inhibiting ECM degradation and inflammatory cytokine production in vivo.

### miR-203a-5p was sponged by circ-NFKB1

**Fig. 6:**
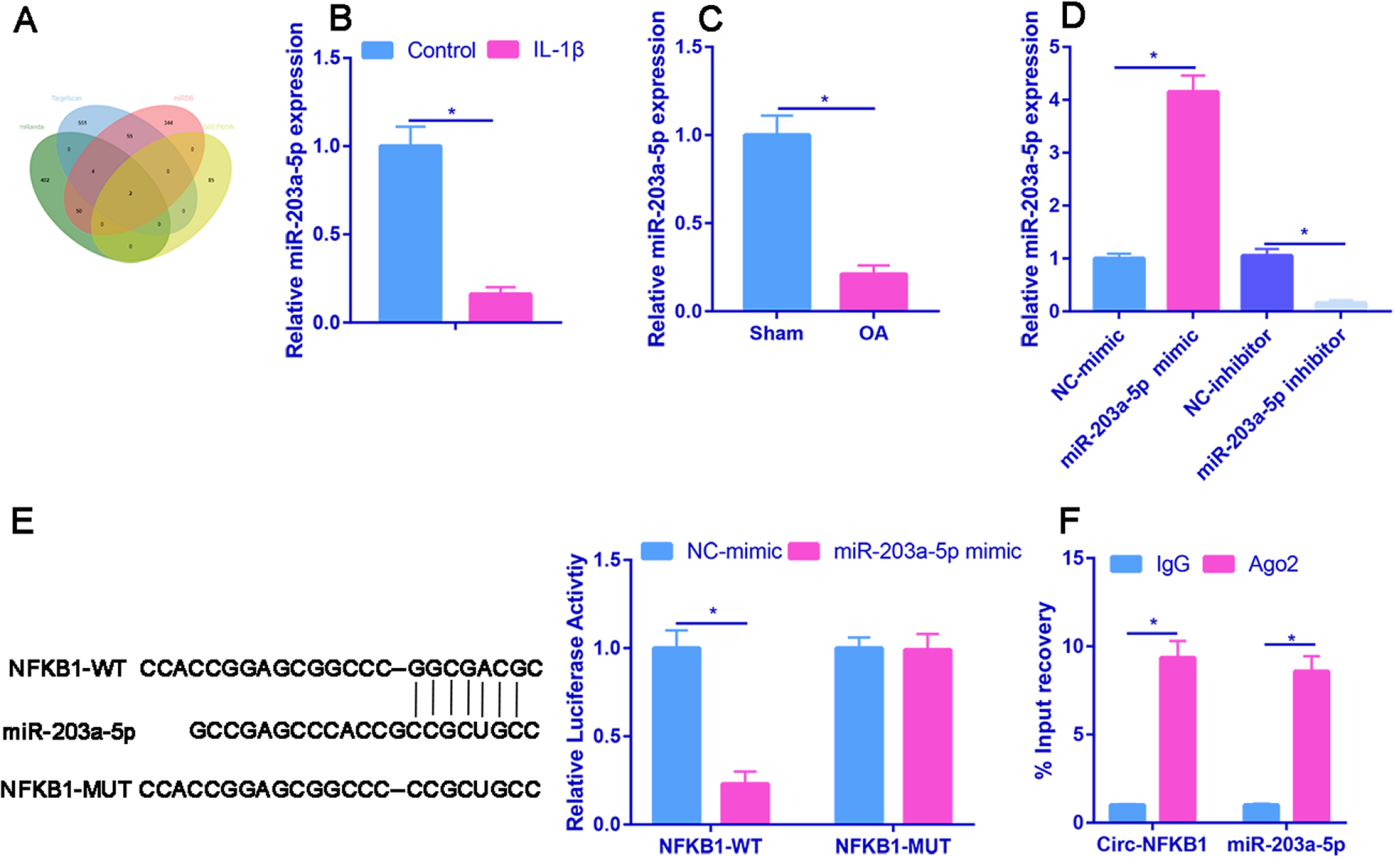
circ-NFKB1 acts as a sponge for miR-374a-3p. **A** Venn diagram depicting overlapping miRNAs across the three databases. **B** RT-qPCR was conducted to detect the relative expression of miR-203a-5p in IL-β-induced chondrocytes. **C** Relative expression of miR-203a-5p in sham and OA cartilage tissue. **D** The miR-203a-5p expression in miR-203a-5p mimic or miR-203a-5p inhibitor groups. **E** The predicted binding sites between miR-203a-5p and NFKB1 and luciferase reporter assay results. **F** The interaction between NFKB1 and miR-203a-5p was confirmed by RIP assay. *P < 0.05

We predicted circ-NFKB1 targeted miRNAs through miRanda, Targetscan and miRDB database. Moreover, these three databases were intersected with differentially expressed miRNAs through GSE79258 dataset. Venn diagram revealed that circ-NFKB1 may interact with miR-203a-5p (Fig. 6A). As shown in Fig. 6B, miR-203a-5p was significantly downregulated in IL-1β treated chondrocytes and OA samples. Real-time PCR analysis showed that miR-203a-5p mimic can increase miR-203a-5p mRNA level, while miR-203a-5p inhibitor can decrease miR-203a-5p mRNA level (Fig. 6C).

Applying CircInteractome software, we identified that circ-NFKB1 has potential binding sites for miR-203a-5p (Fig. 6D). In addition, dual-luciferase reporter gene assay manifested that the miR-203a-5p mimic markedly downregulated luciferase activity in the wild-type NFKB1 group, but exhibited no significant effect in the mutant-type NFKB1 group (Fig. 6E). Furthermore, RIP assay elucidated that circ-NFKB1 and miR-203a-5p expression was significantly enriched in RNA induced silencing complex (RISC) immunoprecipitated by anti-Ago2 rather than anti-IgG (Fig. 6F). Generally, miR-203a-5p could act as a meaningful target of circ-NFKB1.

### ERBB4 is a direct target of miR-203a-5p

**Fig. 7:**
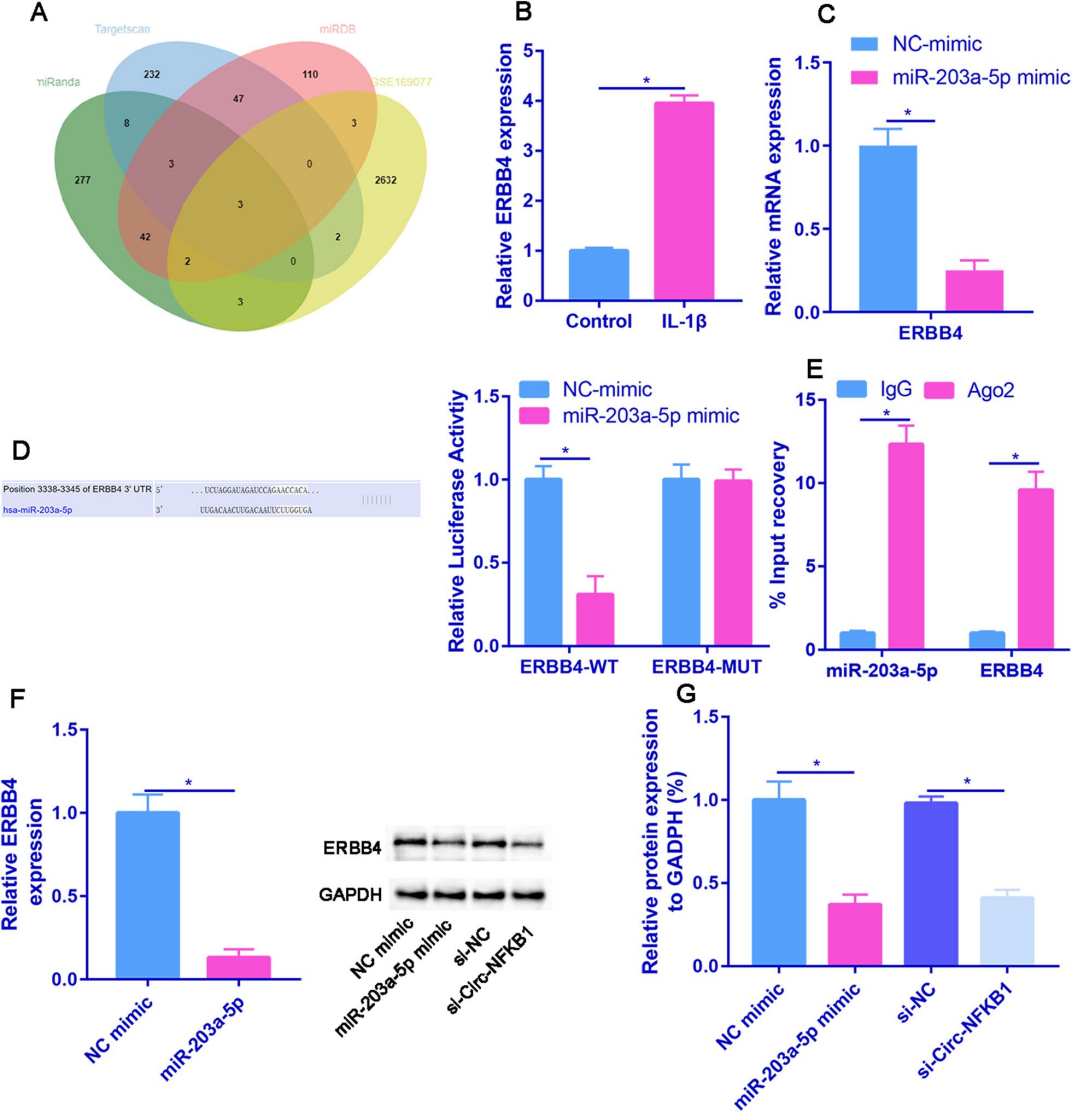
ERBB4 is a direct target of miR-203a-5p. **A** ERBB4 was predicted by intersection of miRanda, Targetscan, miRDB and GSE169077 using the Venn diagram. **B** The expression of candidate mRNAs was measured in chondrocytes transfected miR-203a-5p mimics. **C** RT-qPCR analysis was used to evaluate ERBB4 expression in IL-β-stimulated chondrocytes. **D** Luciferase reporter assay was performed to assess the binding capacity between miR-203a-5p and ERBB4. **E** The binding capacity between miR-203a-5p and ERBB4 was verified by RIP assay. **F** Effects of miR-203a-5p overexpression on the mRNA expression of ERBB4. **F** Effects of miR-203a-5p overexpression or circ-NFKB1 knockdown on the protein expression of ERBB4. *P < 0.05

Figure 7A demonstrates the intersection of three databases (miRanda, Targetscan, and miRDB) and GSE169077, revealing one potential miRNA-mRNA interaction: ERBB4. Furthermore, we assessed the expression of ERBB4 in chondrocytes transfected with NC-mimic or miR-203a-5p mimic, and our results indicated that miR-203a-5p mimics caused the most significant decline in ERBB4 levels (Fig. 7B). Additionally, we observed a significant increase in ERBB4 expression in IL-1β-stimulated chondrocytes (Fig. 7C). Figure 7D showed that miR-203a-5p contains the binding site on the 3′-UTR of ERBB4, and luciferase activity assay indicated that overexpression of miR-203a-5p significantly decreased the luciferase activity of ERBB4-WT vectors but had no obvious effect on the luciferase activity of ERBB4-MUT vectors. We also discovered that both miR-203a-5p and ERBB4 were co-immunoprecipitated by anti-Ago2 but not anti-IgG (Fig. 7E). Moreover, the mRNA and protein levels of ERBB4 were significantly reduced by miR-203a-5p mimics or circ-NFKB1 knockdown (Fig. 7F and G). In conclusion, we established that ERBB4 is a direct target of miR-203a-5p.

### Circ-NFKB1 exacerbates OA development by regulating ERBB4

**Fig. 8:**
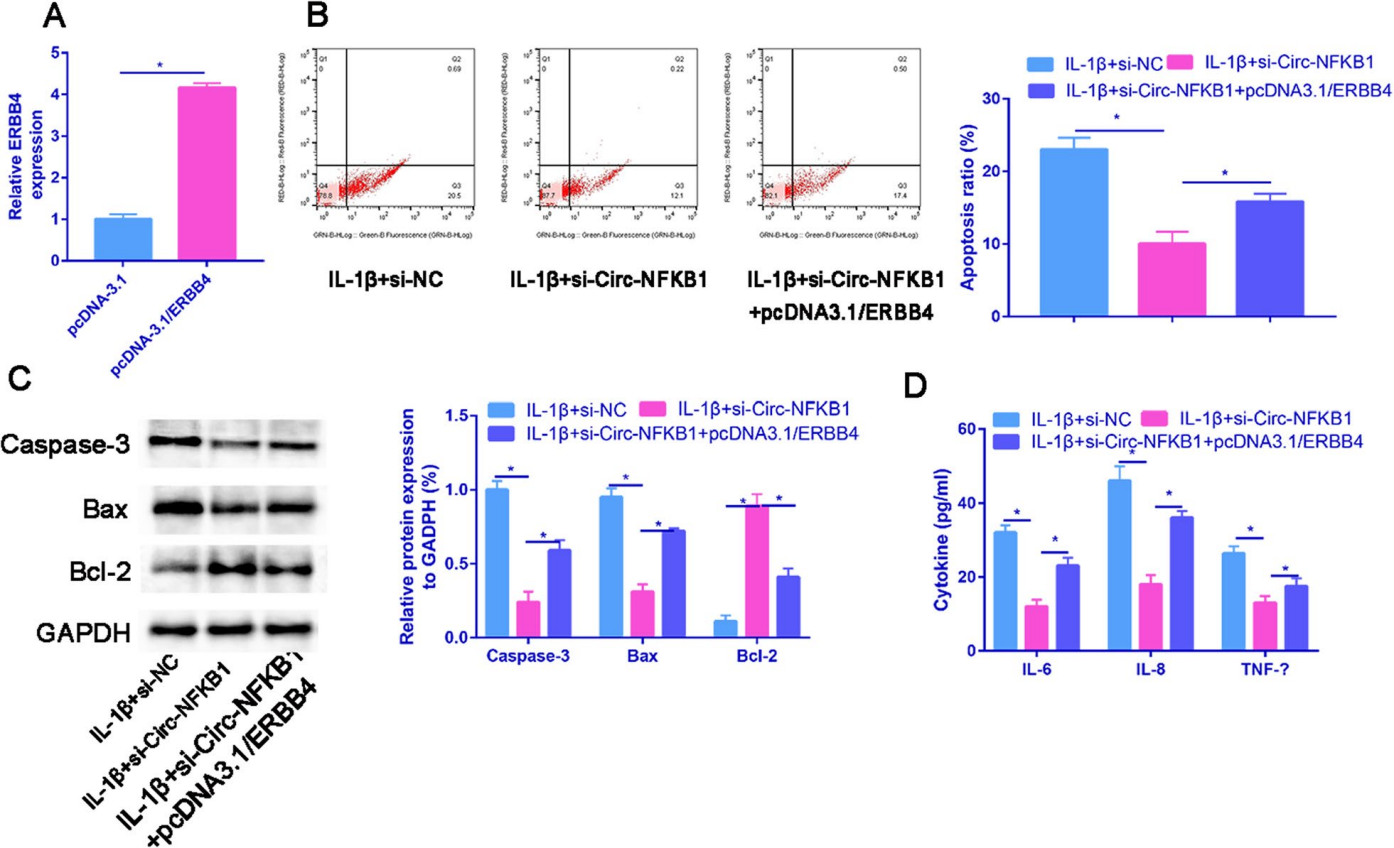
Overexpression of ERBB4 could partially reverse the inhibitory effects of circ-NFKB1 knockdown by siRNA of circ-NFKB1 on IL-1β induced chondrocytes apoptosis. **A** The overexpression efficiency of ERBB4 was determined by RT-qPCR. **B** Flow cytometry was applied to measure the apoptosis ratio in transfected chondrocytes. **C** Caspase-3, Bax and Bcl-2 protein expressed that detected by western blot analysis. **D** The concentration of inflammatory cytokines was measured by ELISA in transfected chondrocytes. *P < 0.05

Firstly, we detected the overexpression efficiency of ERBB4 using RT-PCR, which showed a significant increase in ERBB4 expression in chondrocytes transfected with pcDNA3.1/ERBB4 (Fig. 8A). As illustrated in Fig. 8B, ERBB4 overexpression significantly reversed the inhibition of apoptosis caused by circ-NFKB1 silencing in IL-1β-induced chondrocytes. Additionally, in IL-1β-treated chondrocytes, ERBB4 upregulation dramatically offset the decreased protein level of Caspase-3 and Bax, as well as the increased protein level of Bcl-2 induced by circ-NFKB1 knockdown (Fig. 8C). Furthermore, our findings suggested that ERBB4 upregulation overturned the suppressive effects of circ-NFKB1 downregulation on the production of inflammatory cytokines (IL-6, IL-8, and TNF-α; Fig. 8D). Overall, these results demonstrate that circ-NFKB1 contributes to OA development by modulating ERBB4.

## Discussion

The dysregulation of circRNAs is frequently observed in numerous human diseases, including OA. Nevertheless, the implications of circRNA in IL-1β induced chondrocytes injury are rarely exposed. Our study identified that circ-NFKB1 was significantly upregulated in OA sample and IL-1β treated chondrocytes. Furthermore, silencing circ-NFKB1 alleviating the IL-1β induced chondrocytes apoptosis, thus further retard OA progression. Circ-NFKB1 targeted miR-203a-5p to modulate the ERBB4 expression. ERBB4 overexpression greatly offset the functional effects of circ-NFKB1 downregulation among the IL-1β-treated chondrocytes.

With the advancement in the study of genetics and the development of advanced biotechnologies such as RNA sequencing and bioinformatics learning methods, an increasing number of non-coding RNAs have been identified, and their functional roles are gaining more attention through extensive research.

Recent studies have shown that circRNA plays a potential role in OA treatment. circRNA plays crucial biological roles, such as acting as a "sponge" for miRNA, serving as a functional protein decoy, and facilitating translation, and their malfunction is linked to the onset and advancement of diverse human diseases. For example, circ_0002715 promotes the development of osteoarthritis through regulating LXN by sponging miR-127-5p [23]. Our research aims to focus on whether circ-NFKB1 plays an important role in the development of OA by targeting miRNA.

Firstly, we performed bioinformatic analysis through GEO database and found that circ-NFKB1 was significantly upregulated in IL-1β treated chondrocytes and OA sample. Moreover, silencing circ-NFKB1 could partially reduce the IL-1β induced apoptosis of chondrocytes. The results demonstrate the significant contribution of circ-NFKB1 in the onset and progression of OA. To realize the potential crosstalk between circ-NFKB1 and ERBB4, we hypothesized that circ-NFKB1 served as a molecular sponge of a certain miRNA to regulate ERBB4 expression. With the help of bioinformatics tools, miR-203a-5p was revealed to harbor binding sites with both circ-NFKB1 and ERBB4. MiR-203a-5p deregulation has frequently been reported to be associated with cancer progression, and miR-203a-5p widely played anti-cancer effects by sequestering downstream targeted oncogenes in diverse cancers [25, 26]. However, the implication of miR-203a-5p in chondrocytes injury has yet to be expounded. Our study has been the pioneer in investigating the functional effects of miR-203a-5p in IL-1β induced chondrocytes injury. Our findings indicate that miR-203a-5p expression was downregulated in IL-1β induced chondrocytes. Thus, we defined that circ-NFKB1 knockdown attenuated the apoptosis of chondrocytes induced by IL-1β by upregulating miR-203a-5p.

ERBB4 is involved in multiple signaling pathways such as PI3K-Akt signaling pathway. Xie et al. [27] revealed that ERBB4, HSPA4L and ST5, were confirmed to be regulated by taraxasterol induced OA model. The results are in keeping with our study. In our study, we confirmed that ERBB4 is a downstream gene of miR-203a-5p and overexpression ERBB4 could increase the apoptosis of chondrocytes that induced by si-circ-NFKB1.

It is worth noting that limitations exist in our present work. For example, oxidative stress has been reported to be activated after chondrocyte injury. Also, evidence regarding the role of circ-NFKB1 on oxidative stress in chondrocyte injury is lacking in our study. Furthermore, more downstream molecules of circ-NFKB1, aside from ERBB4, need further exploration.

## Conclusion

In summary, this work is the first to determine the new molecular mechanism of the circ-NFKB1/miR-203a-5p/ERBB4 pathway in OA progression. More specifically, circ-NFKB1 suppressed miR-203a-5p expression by sponging miR-203a-5p and enhanced ERBB4 expression to promote chondrocyte apoptosis and OA progression.

## Data Availability

Not applicable.

## Abbreviations

OA: Osteoarthritis
IL-1β: Interleukin-1β
GEO: Gene Expression Omibus
lncRNAs: Long non-coding RNA
miRNAs: Small RNA
circRNAs: Circular RNA
PVDF: Polyvinylidene difluoride
RIPA: Radioimmunoprecipitation
RIP: RNA immunoprecipitation
FITC: Fluorescein isothiocyanate
PI: Propidium iodide
OD: Optical density
